# Cyst of the canal of Nuck mimicking inguinal hernia

**DOI:** 10.1016/j.ijscr.2018.09.053

**Published:** 2018-10-10

**Authors:** Uğur Topal, Ahmet Gökhan Sarıtaş, Abdullah Ülkü, Atılgan Tolga Akçam, Figen Doran

**Affiliations:** a Çukurova University, Department of General Surgery, Adana, Turkey; b Çukurova University, Department of Pathology, Adana, Turkey

**Keywords:** Canal of Nuck, Female hydrocele, Inguinal hernia

## Abstract

**Introduction:**

Coley, in 1892, reported 14 cases of a hydrocele in women. He described this “affection” as being “too rare an anomaly to deserve consideration. The rarity of this finding continues to be described in more current literature of 400 cases.

**Case presentation:**

42-year-old woman presented to the clinic with a palpable mass in her left inguinal region. On physical examination, a soft-consistency, mobile mass of about 4 cm in size was seen in the left inguinal region. The cystic lesion which was seen to be originated from the inguinal canal was excised in the exploration made by suspending the round ligament by passing through the anatomical folds with the incision made from the left inguinal region. The defect was repaired with prolene mesh after high ligation. Patient was discharged on the 1st postoperative day.

**Discussion:**

In women, a round ligament is attached to the uterus close to the origin of the fallopian tubes, and the extension of the parietal peritoneum follows the round ligament as it passes to the inguinal canal through the internal ring. Hydroceles of the canal of Nuck were not conclusively diagnosed until surgery was performed on a suspected inguinal hernia. The treatment of Nuck canale hydroceles are surgery. Ligating the prosessus vaginalis and excision of the cyst in surgical treatment will prevent recurrences.

**Conclusions:**

Nuck canal cysts should be considered in the differential diagnosis of cases of female patient's complaints of swelling in the inguinal region.

## Highlights

• This is a rare diagnosis, and the differential diagnosis often includes more common causes of inguinal masses.
• In this document, we aimed to present the rare case of a Nuck canal cyst.

## 1 Introduction

Coley, in 1892, reported 14 cases of a hydrocele in women. He described this “affection” as being “too rare an anomalyto deserve consideration” [1]. During embryological development of a female fetus, round ligament of the uterus descends down to the ipsilateral labia majora through the inguinal canal. A peritoneal fold also descends along with the round ligament, which is known as canal of nuck. It was first described by Dutch anatomist, Anton Nuck in 1691. It usually gets obliterated by birth or during early infancy but if this communication remains patent, it may lead to development of an indirect inguinal hernia or hydrocele. In surgical practice, congenital hydrocele or hernia cases are usually seen in a male child and it is uncommon in females [2]. The rarity of this finding continues to be described in more current literature of 400 cases [3]. The cysts seldom exceed3cm in length with a diameter of 0.3*0.5 cm [4]. We aimed to assist the differential diagnosis and to contribute to the literature by presenting the Nuck Channel hydrocele case, which should be considered in the differential diagnosis of inguinal duct swelling and which there is few examples of in the literature.

## 2 Case report

A 42-year-old woman presented to the clinic with a palpable mass in her left inguinal region which was noticed 1 month prior. The mass had not been present in infancy oradolescence. History of trauma and operations were not found in the patient's history. There was a cyst aspiration story from 2 months ago. On physical examination, a soft-consistency, mobile mass of about 4 cm in size was seen in the left inguinal region. During the Valsalva maneuver, the mass did not change in size and shape. The patient's laboratory findings (complete blood count, urinalysis, blood biochemistry) were within the normal range. Ultrasonography revealed a hypoechoic cystic mass with a size of 40 × 50 mm in the left inguinal area without any vascular flow and no peristalsis (Picture 1).

**Picture 1:**
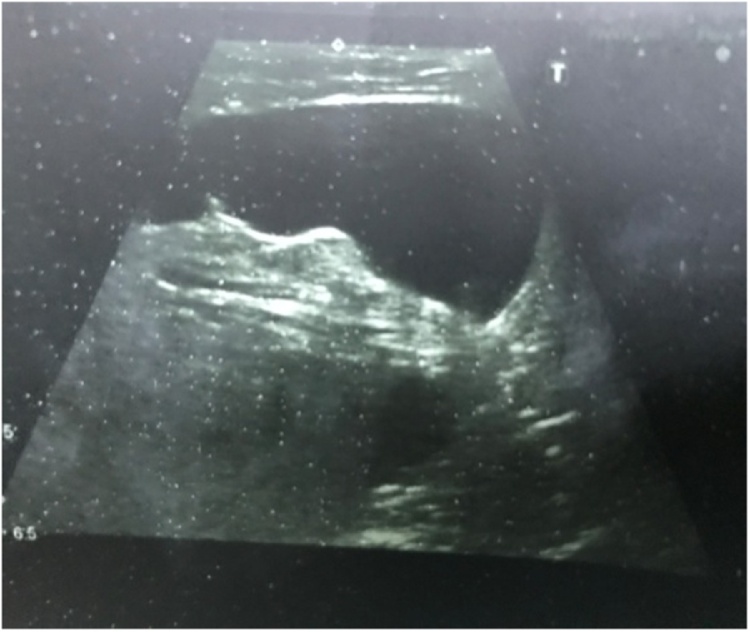
Cyst of the Canal of Nuck of Ultrasonography view.

Abdominal magnetic resonance imaging (MRI) was performed to examine the communication between the cystic mass and peritoneal cavity, and the precise anatomy around the cystic mass.

It was found that the cystic mass in the inguinal canal included thin septa, and hydrocele of the canal of Nuck was suspected because of the low and high signal intensities observed on the T1- and T2-weighted images, respectively. Only the wall and septa were contrast-enhanced. The cystic lesion which was seen to be originated from the inguinal canal was excised in the exploration made by suspending the round ligament by passing through the anatomical folds with the incision made from the left inguinal region (Picture 2, Picture 3).The defect was repaired with prolene mesh after high ligation. Histopathologic examination was evaluated as Simple cystic structure with cubic epithelium (Picture 4). Patient was discharged on the 1 st postoperative day. The patient provided written consent to utilize her medical record with no patient identifiers.

**Picture 2:**
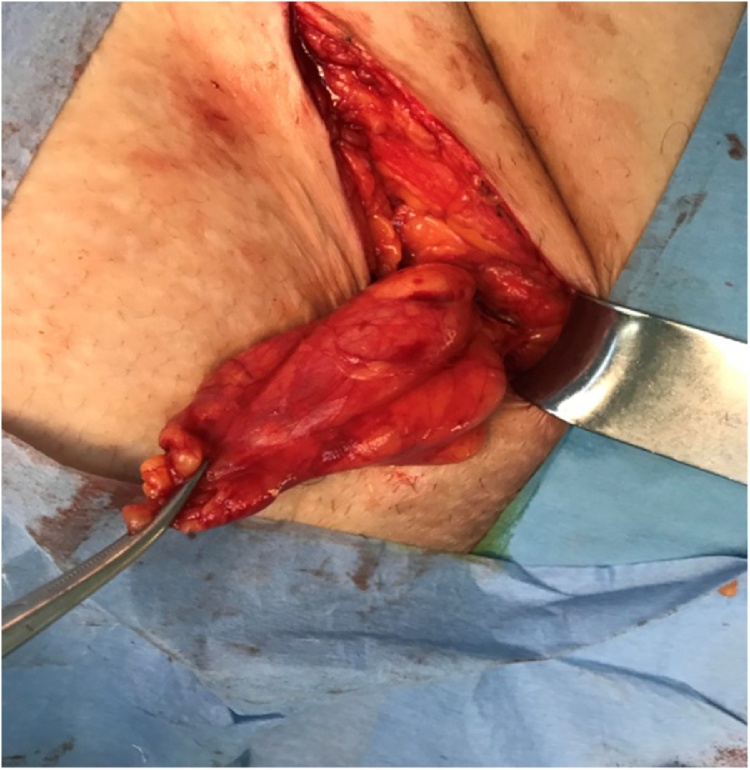
Intraoperatively view.

**Picture 3:**
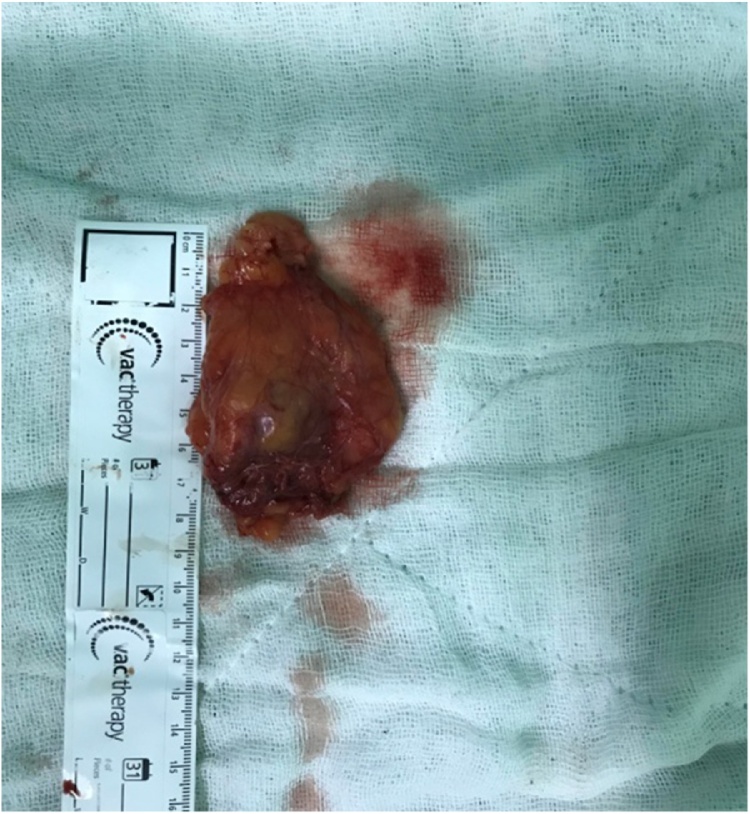
Pathologic specimen.

**Picture 4:**
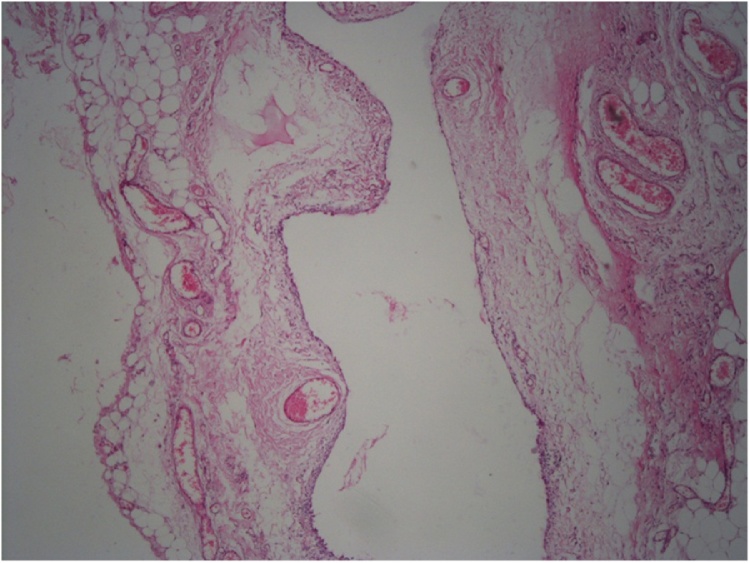
Simple cystic structure with cubic epithelium, Hemotok silen eozin.

## 3 Discussion

In women, a round ligament is attached to the uterus close to the origin of the fallopian tubes, and the extension of the parietal peritoneum follows the round ligament as it passes to the inguinal canal through the internal ring. This evagination of the parietal peritoneum, named the canal of Nuck, is known as the female counterpart of the processus vaginalis in men. The cystic enlargement is probably due to the imbalance of the secretion and absorption of the secretory membrane that covers the processus vaginalis. This imbalance may be caused by a change in lymphatic drainage as a result of trauma or infection, although most cases are idiopathic [5]. There are three types of a hydrocele of canal of Nuck. The most common type is one with no communication with peritoneal cavity forming an encysted hydrocele along the tract of descent, from the inguinal ring to the vulva. Second type results when there is a persistent communication with the peritoneal cavity. A third type is a combination of the two as a result of the inguinal ring constricting the hydrocele like a belt so that part is communicating and part is enclosed, giving this the name of hour glass type [6]. Cystic groin masses in women are rare. Clinically, masses in the groin are often diagnosed as hernias or lymphadenopathies. Differential diagnosis based on clinical findings alone is difficult because the abnormalities have similar characteristics.

The majority of the reported cases of hydroceles of the canal of Nuck were not conclusively diagnosed until surgery was performed on a suspected inguinal hernia [7].

Women's hydroceles usually manifest themselves as a painless palpable mass in the groin area. Suspicion may arise if the mass is translucent, doesn't disappear in the supine position -like in our patient- or if it becomes more pronounced with valsalva. Imaging studies may help in pre-operative diagnosis but most of the cases of hydrocele of canal of nuck finally diagnosed on surgical exploration. Cyst of the canal of Nuck demonstrates varied appearances in sonography. In the literature sonographic appearance of hydrocele of the canal of Nuck shows thin walled, well defined, echo free, cystic structure varying from an anechoic, tubular, sausage, dumbbell or comma-shaped, “cyst within a cyst’’ to a multicystic appearance [[5], [6], [7], [8], [9]]. Magnetic resonance imaging may be performed in cases where the diagnosis is suspicious, to determine things such as intraabdominal extension of the mass, and association with other organs. Definitive diagnosis is made by histopathologic examination after excision [9]. The treatment of Nuck canale hydroceles are surgery. Ligating the prosessus vaginalis and excision of the cyst in surgical treatment will prevent recurrences. However, surgical use of synthetic polyproplene mesh in cases of potential recurrence will prevent surgical failure. Cyst aspiration has no place in treatment [10].

On pathological examination, the Nuck duct cyst is defined as a cyst that is filled with light-colored fluid and has smooth muscle tissues on its walls and is laid with single-layer cuboidal mesothelial cells [9].

Nuck canal cysts should be considered in the differential diagnosis of cases of female patient's complaints of swelling in the inguinal region.

## Conflict of interest

No conflicts of interest were declared.

## Sources of funding

We have no supportive funding.

## Ethical approval

I certify that this kind of manuscript does not require ethical approval.

## Consent

Written informed consent for publication of his clinical details and clinical images was obtained from the patient.

## Author contribution

Uğut topal.-Ahmet gökhan sarıtaş study concept, writing the paper, final decision to publish, data collection.

Abdullah ülkü.- study concept, data collection.

Atılgan tolga akçam.- data collection and analysis.

## Guarantor

Ahmet Gökhan Sarıtaş, Abdullah Ülkü.

## Provenance and peer review

Not commissioned, externally peer reviewed.
